# Quantification of regenerative potential in primary human mammary epithelial cells

**DOI:** 10.1242/dev.123554

**Published:** 2015-09-15

**Authors:** Jelena R. Linnemann, Haruko Miura, Lisa K. Meixner, Martin Irmler, Uwe J. Kloos, Benjamin Hirschi, Harald S. Bartsch, Steffen Sass, Johannes Beckers, Fabian J. Theis, Christian Gabka, Karl Sotlar, Christina H. Scheel

**Affiliations:** 1 Institute of Stem Cell Research, Helmholtz Center for Health and Environmental Research Munich, Neuherberg 85764, Germany; 2 Institute of Experimental Genetics, Helmholtz Center Munich, Neuherberg 85764, Germany; 3 Institute of Pathology, Medical School, Ludwig Maximilian University Munich, Munich 80337, Germany; 4 Institute of Computational Biology, Helmholtz Center Munich, Neuherberg 85764, Germany; 5 Department of Experimental Genetics, Technical University Munich, Freising 85354, Germany; 6 Department of Mathematics, Technical University Munich, Garching 85747, Germany; 7 Nymphenburg Clinic for Plastic and Aesthetic Surgery, Munich 80637, Germany

**Keywords:** Mammary stem cell, Primary human mammary epithelial cells, Basal, Luminal, Progenitor cells, Branching morphogenesis, Organoid, Regenerative potential, CD10, Terminal ductal-lobular units, Collagen gel

## Abstract

We present an organoid regeneration assay in which freshly isolated human mammary epithelial cells are cultured in adherent or floating collagen gels, corresponding to a rigid or compliant matrix environment. In both conditions, luminal progenitors form spheres, whereas basal cells generate branched ductal structures. In compliant but not rigid collagen gels, branching ducts form alveoli at their tips, express basal and luminal markers at correct positions, and display contractility, which is required for alveologenesis. Thereby, branched structures generated in compliant collagen gels resemble terminal ductal-lobular units (TDLUs), the functional units of the mammary gland. Using the membrane metallo-endopeptidase CD10 as a surface marker enriches for TDLU formation and reveals the presence of stromal cells within the CD49f^hi^/EpCAM^−^ population. In summary, we describe a defined *in vitro* assay system to quantify cells with regenerative potential and systematically investigate their interaction with the physical environment at distinct steps of morphogenesis.

## Abstract (precis)

**Summary:** An assay in which single, freshly isolated human mammary epithelial cells are cultured in a matrix environment is developed and used to quantify the regenerative potential of human mammary cells.

## INTRODUCTION

The mammary gland (MG) develops from the anlage, a cluster of specified ectodermal cells forming a rudimentary ductal tree before birth (Sternlicht, 2006). Puberty induces outgrowth into an expansive network of ducts, draining the milk-producing, terminal ductal-lobular units (TDLUs) (Brisken and O'Malley, 2010). Extensive proliferation and remodeling during each menstrual cycle and pregnancy, and the ability of single murine mammary epithelial cells (MECs) to reconstitute a functional MG in transplantation assays, suggest the existence of adult mammary stem cells (MaSCs) (Brisken and Duss, 2007; Fridriksdottir et al., 2011; Visvader and Stingl, 2014). However, the presence of MaSCs appears to depend on developmental stage (van Amerongen et al., 2012), and whether homeostasis or regeneration is required (Rios et al., 2014; Van Keymeulen et al., 2011; Wang et al., 2014). Consequently, defining the molecular identity of MaSCs remains an active area of investigation.

Importantly, significant differences in cellular and matrix composition between the mouse and human mammary stroma hamper assessment of human MaSC activity in the mouse (Parmar and Cunha, 2004). Limited *in vivo* growth of human mammary epithelial cells (HMECs) has been achieved by humanization of the mouse fat pad (Proia and Kuperwasser, 2006) or transplantation under the renal capsule (Eirew et al., 2008). Alternatively, the MaSC potential of HMECs has been assessed *in vitro*, but relied on previously cultured cells and support from non-mammary gland derived stromal cells (Dontu et al., 2003; Eirew et al., 2008; Gudjonsson et al., 2002; Stingl et al., 2005). Based on these considerations, we set out to recapitulate major aspects of branching morphogenesis using freshly isolated HMECs.

Importantly, the two major lineages that constitute the MG correspond to the position of cells within the ducts: the inner luminal cells secrete milk into the central lumen during lactation. The outer layer of myoepithelial/basal cells contracts to release milk, and is enveloped by a basement membrane mainly composed of laminins and collagen IV (Muschler and Streuli, 2010). Extracellular matrix components, such as collagen I, surround and support the ductal network.

Guided by these parameters, we developed an organoid assay where single, freshly isolated HMECs, cultured in collagen gels, generate organoids that resemble TDLUs. The TDLU-like organoids form alveolar buds, express multiple lineage markers at correct positions and display contractility, which our assay reveals to be required for alveologenesis. Remarkably, an increase in matrix compliance by switching collagen gels from an adherent, rigid state to free flotation suffices to trigger alveologenesis, emphasizing the importance of physical parameters in directing differentiation of the MG (Bainer and Weaver, 2013; Schedin and Keely, 2011).

In line with the assumption that MaSCs reside in the basal subpopulation, we determined that TDLU-like structure formation is enriched in the CD49f^hi^/EpCAM^−^ population, commonly referred to as basal. However, by performing extreme limiting dilution analysis (ELDA), we identify the membrane metallo-endopeptidase CD10 as a marker to enrich for TDLU-like structure-forming cells and reveal the presence of heterogeneous stromal cells within the CD49f^hi^/EpCAM^−^ population. Together, our data highlight diversity and plasticity of cell populations in the human MG, while revealing robustness of functional and phenotypic qualities in isolated subpopulations.

## RESULTS

### Identification of culture conditions that promote generation of TDLU-like structures

**Fig. 1.**
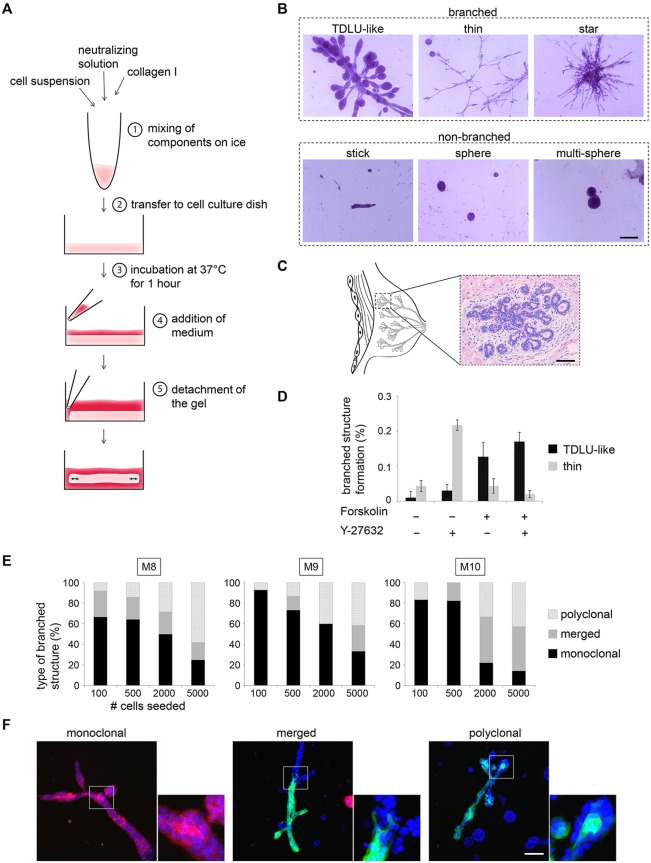
**Identification of culture conditions promoting generation of TDLU-like structures.** (A) Experimental setup: floating collagen gels. (B) Bright-field microscopy: carmine-stained representative images of different types of branched and non-branched structures (donor M8). Scale bar: 200 µm. (C) Bright-field microscopy: Hematoxylin and Eosin-stained section of a terminal ductal lobular unit (TDLU) from a healthy woman. Scale bar: 100 µm. (D) Improvement of culture conditions: one-time treatment with 3 µM Y-27632 at day 0 of culture and continuous treatment with 10 µM forskolin (14 days of culture). Star-like branched structures were not detected. *n*=3 gels/condition. Structure formation per 100 seeded cells is shown (donor M8). Data are mean±s.d. (E) Quantification of monoclonal, merged and polyclonal structures formed by eGFP, mCherry and unlabeled passage 1 cells (donors M8, M9, M10) in floating collagen I gels: 100, 500, 2000 and 5000 cells were seeded per well (24-well plate). Monoclonal: complete structure eGFP or mCherry positive. Merged: monoclonal structure merged with second structure. Polyclonal: eGFP/mCherry-positive and -negative areas. *n*≥9 eGFP/mCherry-positive structures/condition. (F) Confocal microscopy: representative images of monoclonal, merged and polyclonal structures as defined in E. Scale bar: 100 µm.

To recapitulate morphogenesis in a 3D-culture system, we chose collagen type I as a substrate, because it constitutes a main component of extracellular matrix in the human MG and provides defined properties that can be modified to model different microenvironments. Moreover, we built on observations that a breast carcinoma cell line generated tubular structures in freely floating collagen gels (Fig. 1A; see Materials and Methods) (Wozniak and Keely, 2005). Within 10-12 days, freshly isolated single-cell suspensions of HMECs cultured in floating collagen gels gave rise to multicellular structures that were subdivided into three types of branched (TDLU-like, thin, star) and three types of non-branched structures (stick, sphere, multi-sphere, Fig. 1B). The TDLU-like structures displayed side-branched ducts with rounded, alveolar tips, similar to the morphology of TDLUs *in situ* (Fig. 1B,C). TDLUs are histological units of the breast consisting of a cluster of up to 100 alveoli, i.e. round buds at the tips of branches. Because TDLUs are the functional units of the MG (Anderson et al., 1998), we focused on characterizing cells and conditions enabling their formation.

As only about 1 in 2000 primary HMECs plated into the gels was able to generate branched-type structures (Fig. 1D), we sought to improve culture conditions. Recent studies have shown that inhibitors of Rho-associated kinase (ROCK) increase colony formation in 2D and 3D culture, and allow for the acquisition of regenerative capacity by mouse MECs (Guo et al., 2012; Makarem et al., 2013; Prater et al., 2014). Thus, ROCK-inhibitor Y-27632 was added to the growth medium upon plating of freshly dissociated cells to promote initial survival. After a period of 5 days, the growth medium was replaced and the ROCK inhibitor removed. We observed that treatment with 3 µM of the ROCK inhibitor Y-27632 increased branched structure formation by approximately fivefold (Fig. 1D; supplementary material Fig. S1A). Similar observations were made with thiazovivin, another ROCK inhibitor (supplementary material Fig. S1A). Importantly, higher concentrations of ROCK inhibitors led to formation of star-like agglomerations and loss of TDLU-like structures (supplementary material Fig. S1B). Continuous treatment with Y-27632 after 5 days of initial culture resulted in dissolution of cell-cell adhesion, thereby perturbing morphogenesis (supplementary material Fig. S1D).

Though addition of ROCK inhibitors increased branched structure formation, the structures were thin in diameter with few alveoli (Fig. 1D). To increase alveologenesis, we added forskolin to the growth medium, an agonist of adenylyl cyclase (Fradkin et al., 1982). Compounds raising cAMP levels are commonly used for epithelial cultures (Stampfer, 1982), and promote polarization and lumen formation in 3D culture (Nedvetsky et al., 2012). Indeed, addition of 10 µM forskolin promoted the formation of TDLU-like structures by ∼12-fold, while overall branched structure-forming potential was increased threefold (Fig. 1D; supplementary material Fig. S1A,B). Formation of non-branched structures (mostly spheres) was only slightly increased (∼1.5-fold; supplementary material Fig. S1C). Together, these results indicated that forskolin promotes the formation of alveolar buds in branched structures. In conclusion, treatment with 3 µM Y-27632 during initial establishment of the organoid cultures and continuous treatment with 10 µM forskolin was used as standard condition. Thereby, the predominant types of structures generated by freshly isolated HMECs were TDLU-like branched structures and spheres.

Matrigel, a basement membrane-protein mixture, is a commonly used substrate for the 3D culture of mammary epithelial cells (Benton et al., 2014; Mailleux et al., 2008). For comparisons with cellular behavior in collagen gels, we seeded HMECs into matrigel. Strikingly, matrigel did not support the growth of freshly isolated HMECs (data not shown). Indeed, it has been argued that primary HMECs need to be established in 2D-culture before cultivation in matrigel (Dontu et al., 2003) or require support by stromal cells (Eirew et al., 2008).

### Single HMECs give rise to TDLU-like structures

To test for clonality of TDLU-like structures, we labeled a fraction of freshly isolated HMECs with eGFP or mCherry fluorescent protein by lentiviral transduction before plating cells in several concentrations. After 10 (500-5000 cells/well) or 11 days (100 cells/well) of culture, nuclei were stained with DAPI to determine the frequency of clonal (complete overlap of eGFP or mCherry with DAPI) and polyclonal structures (multicolored) by confocal microscopy. In gels containing 100 or 500 cells, the majority of labeled structures showed a complete overlap of eGFP or mCherry with DAPI, suggesting that they were derived from a single cell (Fig. 1E,F). A minority of structures were multicolored, consisting either of monoclonal structures that had merged or contained smaller intermingled patches of cells (polyclonal). The percentage of multicolored structures increased with cell density (2000, 5000 cells/well), suggesting that merging of structures or intermingling of cells is largely an effect of proximity (Fig. 1E). Of note, the ratio of spheres to branched structures increased sharply at 5000 cells plated, suggesting that high cell density impairs the generation of TDLU-like structures (supplementary material Fig. S1E). Together, these observations demonstrate that single HMECs give rise to TDLU-like structures in floating collagen gels when seeded at low densities.

### Maintenance and expansion of TDLU-like structure formation

To test for the presence of HMECs with regenerative capacity over multiple passages, we enzymatically digested collagen gels to yield single-cell suspensions, which were re-plated into floating collagen gels. We observed formation of branched structures over two passages (supplementary material Fig. S2A). Afterwards, 3D-cultured HMECs predominantly generated spheres, suggesting a loss of regenerative, but not proliferative, capacity (data not shown).

**Fig. 2.**
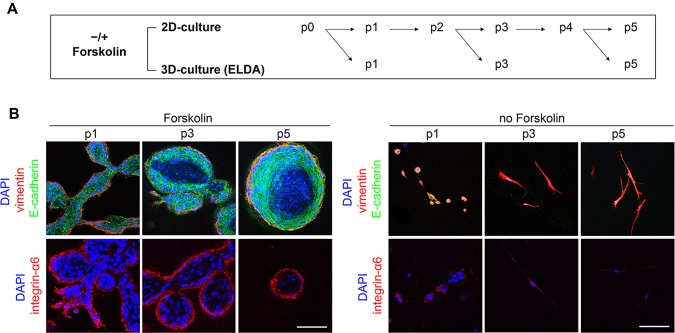
**Maintenance and expansion of TDLU-like structure formation.** (A) Experimental setup: freshly isolated HMECs (donor M4) were cultured in 2D±10 µM forskolin for five passages (p), and transferred to floating collagen I gels in limiting dilution at p1, 3 and 5. (B) Confocal microscopy: representative TDLU-like structures after 2D culture (see A). Vimentin (red), E-cadherin (green), integrin-α6 (red), DAPI (blue). Scale bars: 100 µm. Data are mean with 95% confidence intervals (CIs).

Genetic manipulation is facilitated by cultivation of cells in 2D rather than 3D culture. To test whether the capacity to form TDLU-like structures is maintained in 2D culture, HMECs were cultured on polystyrene cell culture dishes and plated into floating collagen gels at passages 1, 3 and 5 to determine TDLU-like structure-forming units by extreme limiting dilution analysis (ELDA; Fig. 2A) (Hu and Smyth, 2009). In passages 1 and 3, branching potential was comparable, with ∼1/290 and ∼1/250 cells giving rise to a TDLU-like structure, respectively (Fig. 2B, Table 1). However, TDLU-like structure formation dramatically decreased by passage 5. Importantly, forskolin was required for maintenance of an epithelial morphology in 2D culture (supplementary material Fig. S2B). HMECs cultured in 2D without forskolin spontaneously acquired mesenchymal attributes, as evidenced by acquisition of front-to-back polarization, downregulation of E-cadherin expression at the protein level and upregulation of mesenchymal markers at the protein and transcript levels (supplementary material Fig. S2B,C). When transferred to floating collagen gels, these mesenchymal HMECs generated only a few loose cell agglomerations (Fig. 2B, Table 1). The transcription factor OVOL2, a negative regulator of mesenchymal genes, has recently been found to be essential for morphogenesis in the mouse MG (Watanabe et al., 2014). Indeed, after passage 1, the expression of *OVOL2* started to decrease dramatically in HMECs cultured without forskolin (supplementary material Fig. S2C). Similar dynamics of repression at the transcript and protein level were observed for *ITGA6*/integrin α6 (CD49f), a cell surface marker for basal and luminal progenitors (supplementary material Fig. S2C,D). Together, these results indicate that upregulation of mesenchymal genes in 2D culture impairs regenerative capacity of HMECs.

**Table 1.**
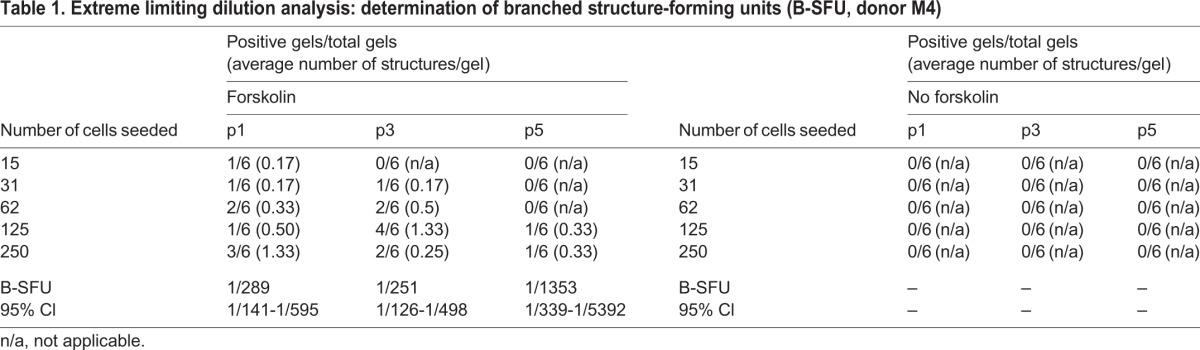
**Extreme limiting dilution analysis: determination of branched structure-forming units (B-SFU, donor M4)**

### Frequency of TDLU-like structure-forming cells varies between donors and is increased by 2D culture

**Fig. 3.**
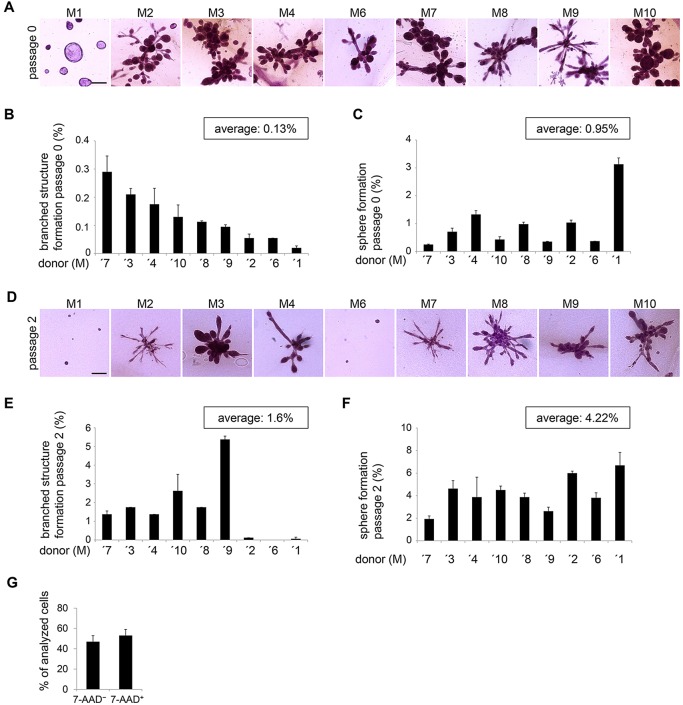
**Frequency of TDLU-like structure-forming cells varies between donors and is increased by 2D culture.** (A) Bright-field microscopy: carmine-stained representative images of TDLU-like structures from freshly isolated cells of nine donors (M1-M4, M6-M10) in floating collagen I gels. Scale bar: 200 µm. (B) TDLU-like structure formation/100 seeded HMECs, 9 days of culture. *n*=2 gels/donor. (C) Sphere formation/100 seeded HMECs, 9 days of culture. *n*=2 gels/donor. (D) Bright-field microscopy: carmine-stained representative images of TDLU-like structures from nine donors (M1-4, M6-M10), 12 days of 2D culture prior transfer to collagen I gels. Scale bar: 200 µm. (E) TDLU-like structure formation/100 seeded HMECs, established in 2D culture (see D), 9 days of culture. *n*=2 gels/donor. (F) Sphere formation/100 seeded HMECs, established in 2D culture, 9 days of culture. *n*=2 gels/donor. Data are shown as mean±s.d. (G) Viability by FACS, using 7-AAD: *n*=10 donors (M1-M10). (B,C,F-H) Data are shown as mean±s.d.

HMECs from individual donors may behave differently due to genetic background, parity and hormonal status (Tanos et al., 2012). To determine the level of reproducibility for cells from different donor tissues, we compared TDLU-like structure formation in nine donors, representing different ages (17-71 years) and parity (0-2; supplementary material Table S1). As expected, structure-forming potential was very heterogeneous (Fig. 3A-C). To quantify representative TDLU-like-structure forming units [b(ranched)-SFU] and sphere-structure forming units (S-SFU), we performed ELDA with a moderately TDLU-like structure- and sphere-forming donor tissue. Thereby, a B-SFU of 1/1005 and a S-SFU of 1/55 were determined (Table 2). In summary, these results show that heterogeneity between donors is reflected by differences in the frequency of cells generating TDLU-like structures and spheres. Within the limited number of donor tissues analyzed, these effects appeared to be independent of age or parity.

**Table 2.**
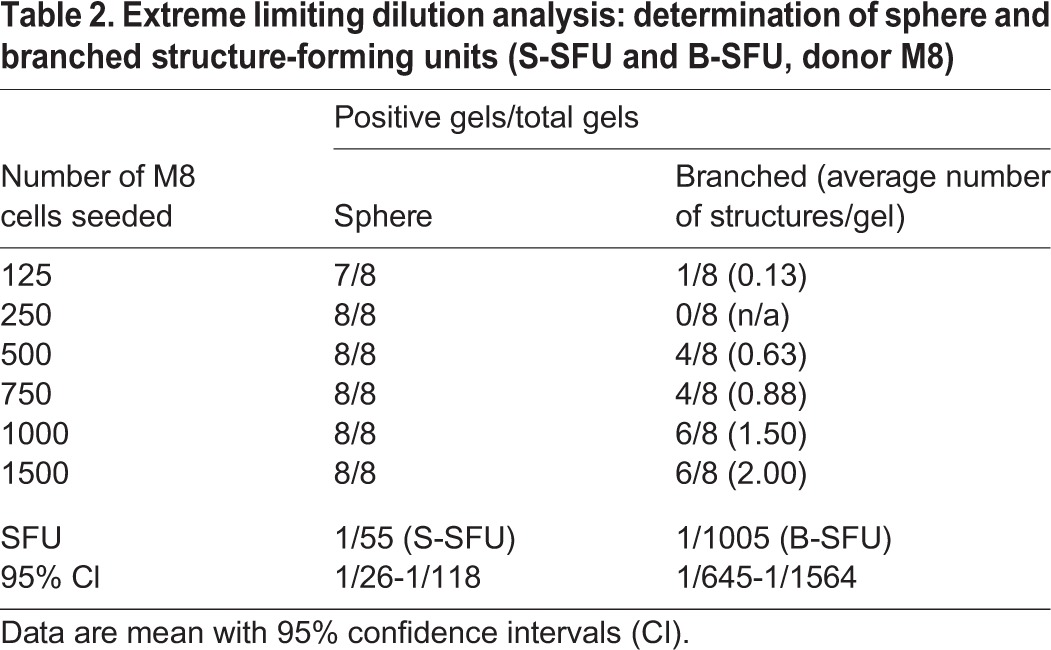
**Extreme limiting dilution analysis: determination of sphere and branched structure-forming units (S-SFU and B-SFU, donor M8)**

Next, we determined whether HMECs from all donors maintained structure-forming ability following establishment in 2D culture. For this purpose, HMECs were established in 2D culture and then transferred to floating collagen gels. Both freshly isolated and 2D cultured HMECs generated TDLU-like structures with similar morphologies, suggesting that short-term 2D culture did not significantly change cell behavior (Fig. 3A,D). Interestingly, we observed that 2D culture increased formation of TDLU-like structures on average by ∼12-fold and formation of spheres by approximately fourfold (Fig. 3E,F). This observation might be largely due to the fact that ∼50% of freshly isolated HMECs are not viable, as determined by 7-AAD labeling (Fig. 3G). Given the overnight processing of tissue required for dissociation, this is expected. Consequently, many of the freshly isolated HMECs plated will not generate structures, resulting in underestimation of TDLU-like structure-forming potential. In addition, these data suggest that either TDLU-like structure-forming cells expand preferentially or, alternatively, some HMECs acquired structure-forming ability *de novo*.

### TDLU-like structure-forming potential is contained within a CD10^+^/CD49f^hi^/EpCAM^−^ basal population

**Fig. 4.**
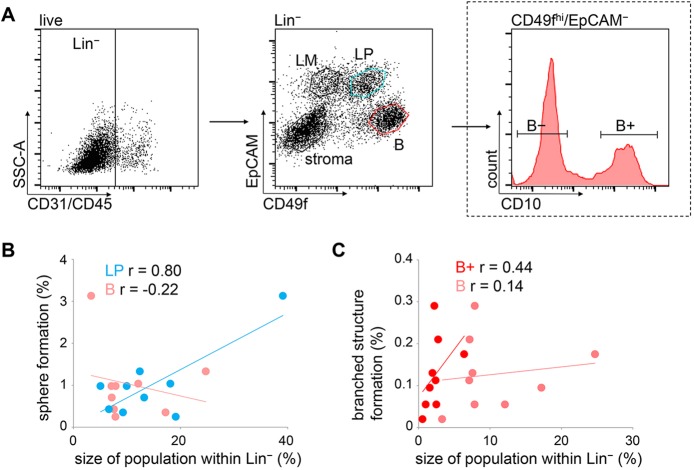
**TDLU-like structure-forming potential is contained within a CD10^+^/CD49f^hi^/EpCAM^−^ basal population.** (A) FACS of freshly isolated HMECs: dead cells (7AAD^−^=live), hematopoietic (CD45^+^) and endothelial cells (CD31^+^) were excluded. EpCAM and CD49f were used to depict the following populations in Lin^−^ (CD45^−^/CD31^−^): stroma (CD49f^−^/EpCAM^­−^), luminal mature (LM, CD49f^−^/EpCAM^+^), luminal progenitors (LP, CD49f^+^/EpCAM^+^) and basal (B, CD49f^hi^/EpCAM^−^). LP and B populations were isolated. The B population was further subdivided into B− (CD10^−^/CD49f^hi^/EpCAM^−^) and B+ (CD10^+^/CD49f^hi^/EpCAM^−^). (B) Linear correlation between sphere formation/100 freshly isolated HMECs and the size of LP within Lin^−^ population (blue dots), or the size of the B population (pink dots). One dot per donor. (C) Linear correlation between TDLU-like structure formation/100 freshly isolated HMECs and the size of B+ within Lin^−^ population (red dots) or the B population (pink dots). One dot per donor. r, correlation coefficient.

MaSCs have been shown to reside within the basal MEC population in the murine MG (Shackleton et al., 2006; Stingl et al., 2006). Therefore, we determined whether the size of the basal and luminal cell population predicts TDLU-like structure and sphere-forming potential in floating collagen gels. Using fluorescence-activated cell sorting (FACS), viable CD45^−^/CD31^−^ (Lin^−^) cells were subdivided based on CD49f and EpCAM expression, as previously described (Fig. 4A) (Eirew et al., 2008). In line with existing data, mature luminal cells (termed LM, CD49f^−^/EpCAM^+^) did not show clonogenic activity in floating collagen gels (data not shown) (Lim et al., 2009). Therefore, we focused on the luminal progenitor (termed LP; CD49f^+^/EpCAM^+^) and basal population (termed B; CD49f^hi^/EpCAM^−^). Thus, respective proportions of LP and B populations within the Lin^−^ compartment of nine donors (supplementary material Fig. S3A) were correlated with organoid formation by freshly isolated bulk HMECs (Fig. 4B,C). We found that sphere formation correlated with the size of the LP population, but not with the size of the B population (Fig. 4B). This observation suggests that spheres predominantly arise from LP. However, neither the size of the LP, nor the size of the B population was predictive of TDLU-like structure formation (Fig. 4C; supplementary material Fig. S3B). Considering that regenerative capacity was shown to reside within the CD49f^hi^/EpCAM^−^ population (Eirew et al., 2008; Lim et al., 2009), we concluded that our observations were probably due to heterogeneity within the B population.

To discern cells with regenerative capacity within the B population, we analyzed expression of the cell surface metalloendopeptidase CD10, which was previously suggested as a potential MaSC marker (Bachelard-Cascales et al., 2010). Two distinct subpopulations were found within the B population; the majority of cells were CD10^−^ (referred to as B−) and a smaller subset was CD10^+^ (referred to as B+, Fig. 4A). CD10^+^ cells were also found among the stromal, LM and LP populations. However, TDLU-like structure formation correlated better with the size of the B+ population than with the percentage of CD10^+^ cells within these other populations (Fig. 4C; supplementary material Fig. S3C). To determine whether CD10 expression within the B population enriches for branching potential, sorted B+, B−, B and LP cell populations were plated in floating collagen gels for ELDA. Indeed, B-SFUs were enriched approximately sevenfold in the B+ population over the B population and ∼30-fold over the B− population (Table 3).

**Table 3.**
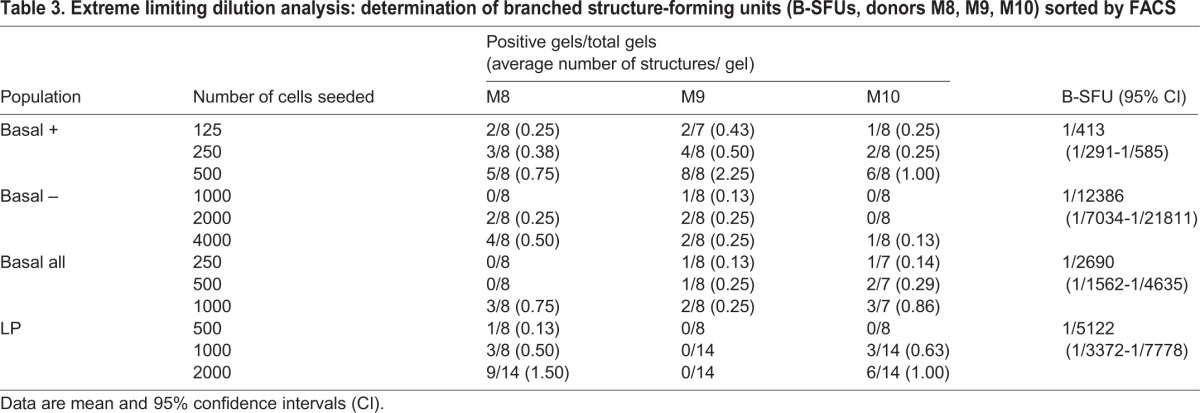
**Extreme limiting dilution analysis: determination of branched structure-forming units (B-SFUs, donors M8, M9, M10) sorted by FACS**

### CD49f^+^/EpCAM^+^ luminal progenitor cells predominantly form spheres in floating collagen gels

LP cells from donor M9 gave rise to spheres, but did not generate any branched structures, as determined by ELDA. Interestingly, LP cells from donor M8 and M10 displayed limited TDLU-like structure-forming ability (Table 3; supplementary material Fig. S3D), which could not be explained by contamination with other cells during the sorting procedure (supplementary material Fig. S3E). These data suggest that the LP population of some donors is able to acquire branching ability.

Plasticity of LP cells has been described before: when transplanted under the renal capsule or into a humanized fat pad of immune compromised mice, human LP cells can give rise to structures containing both luminal and basal cells (Keller et al., 2010; Shehata et al., 2012). However, confocal microscopy revealed that in contrast to TDLU-like structures arising from B+ cells, both spheres and TDLU-like structures were strongly positive for Cytokeratin (CK) 8/18, a marker of luminal cells (supplementary material Fig. S3F). However, in a few cases, we observed CK8/18-negative, vimentin-positive areas that appeared disordered and invasive. By contrast, vimentin stained mostly cells in a basal position in TDLU-like structures arising from B+ cells, which, in a few instances, contained CK8/18-positive areas in a luminal position. Together, these results raise the issue of whether plasticity, when observed in LP cells, is representative of normal regeneration or of illicit dedifferentiation.

### CD10 staining reveals a stromal component within the CD49f^hi^/EpCAM^−^ population

**Fig. 5.**
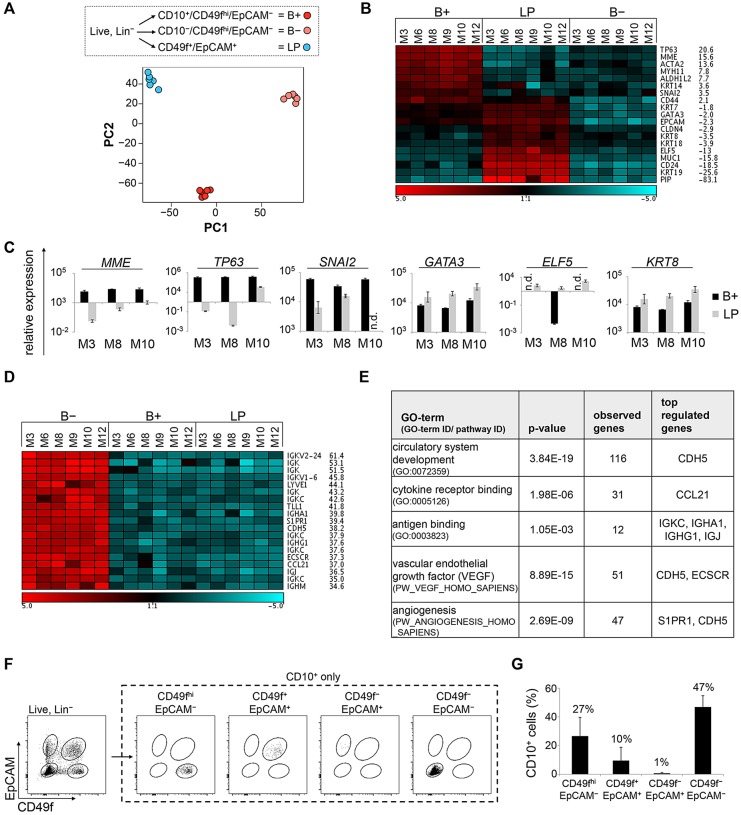
**CD10 staining reveals a stromal component within the CD49f^hi^/EpCAM^−^ population.** (A) Gene expression profiling: RNA for microarray analysis derived from three subpopulations (B+, B− and LP, as indicated) using freshly isolated HMECs from six donors (M3, M6, M8, M9, M10, M12). Unsupervised clustering of all samples was followed by principal component analysis (PCA). (B) Heatmap: expression values of up- and downregulated luminal and basal genes in all samples. Fold-change of B+ versus LP expression levels. Red (high) and blue (low) indicates log2 expression values. Scale bar is in log2. (C) RT-PCR: *MME*/*CD10*, *TP63*, *SNAI2*, *GATA3*, *ELF5* and *KRT8* mRNA expression in B+ and LP cells. *n*=3. (D) Heatmap: expression values of the top 20 significantly (FDR<10%) upregulated genes in B− samples versus B+ samples with corresponding fold-changes. Red (high) and blue (low) indicates log2 expression values. Scale bar is in log2. (E) GO term analyses: selected significantly enriched terms (*P*<0.01) associated with genes differentially regulated between B− and B+ populations (FDR<10%, FC>3×). (F) Representative flow cytometry analysis showing CD10^+^ fraction within the four populations defined by CD49f/EpCAM. (G) Quantification of F. Average of 10 donors (M1-M10). n.d., not detectable. (C,G) Data are shown as mean±s.d.

To assess differences between B− and B+ cells at the phenotypic level, we performed gene expression profiling. For this purpose, we separated freshly isolated cells from six donors of various age and parity into B+, B− and LP populations by FACS (see Fig. 4A; supplementary material Table S1). Principal component analysis (PCA) of global gene expression revealed three distinct clusters corresponding to these different populations (Fig. 5A). Thus, although sizes of the B+, B− and LP populations vary greatly between different donors (supplementary material Fig. S3A), isolated populations cluster tightly across donors at the transcriptional level. In conclusion, robustness in function, i.e. structure formation, is reflected at the transcriptional level. Together, these results support the applicability of employing cell surface markers to isolate distinct subpopulations from primary HMECs to determine regenerative potential.

PCA confirmed that B− and B+ cells represent distinct populations. To understand the cellular identity of these populations, we compared transcript levels of luminal and basal cell-fate determinants (Fig. 5B). As expected, basal genes (such as *TP63* and *ACTA2*) were strongly upregulated in B+ compared with LP cells. Conversely, luminal genes (such as *KRT19*, *MUC1*, *ELF5*) were highly upregulated in LP cells compared with B+ cells. Gene expression levels of *MME* (encoding CD10), *TP63*, *SNAI2*, *GATA3*, *ELF5* and *KRT8* were confirmed by qPCR for three donors, strongly suggesting that B+ cells are basal/myoepithelial (Fig. 5C). Surprisingly, the expression of both basal and luminal cell-fate determinants was low in B− cells compared with B+ and LP cells, calling into question the epithelial identity of these cells (Fig. 5B,C). Indeed, the 20 most highly upregulated transcripts (FDR<10%) in the B− versus B+ population included *IGK* (encoding immunoglobulin chains), *LYVE1* and *CDH5* (encoding VE-cadherin), indicative of B cells, T cells, as well as lymph- and vascular-endothelial cells (Fig. 5D). In support of these data, GO-term analysis revealed groups of genes associated with circulatory system development, cytokine-receptor binding, antigen binding, VEGF and angiogenesis to be significantly overrepresented within the B− compared with the B+ gene expression profile (Fig. 5E). These results suggested that the CD49f^hi^/EpCAM^−^ population, commonly referred to as basal, contains stromal cells, including hematopoietic and endothelial cells. Importantly, a systematic analysis of cell fate markers in the human MG by immunohistochemistry recently revealed that all cells at basal positions express CD10, supporting our conclusion that the B− population contains non-basal cells (Santagata et al., 2014). CD31 and CD45, as employed in our study, are commonly used markers to exclude endothelial and hematopoietic cells from sorted cell populations. However, it has been shown that certain types of endothelial cells, such as in spleen and kidney capillaries, are negative for CD31 (Pusztaszeri et al., 2006). Moreover, transitional B cells as well as plasmablasts and plasma cells are known to downregulate CD45 (Zikherman et al., 2012).

Thus, using CD10 as a cell-surface marker within the CD49f^hi^/EpCAM^−^ population does not merely enrich regenerative cells within the basal cell population, but rather yields a purified basal population. Importantly, CD10 cannot replace CD49f as a surface marker, because it was also expressed on average in 1% of LM (CD49f^−^/EpCAM^+^), 10% of LP (CD49f^+^/EpCAM^+^) and 47% of stromal cells (CD49f^−^/EpCAM^−^) (Fig. 5F,G).

### Branched structures derived from the B+ population display markers of the luminal lineage

As B+ cells were able to form structures in floating collagen gels that resemble TDLUs *in situ*, we hypothesized that they might give rise to cells of the luminal lineage, analogous to bipotent progenitors or MaSCs. By contrast, we expected LP cells to be mostly restricted to a luminal cell fate.

**Fig. 6.**
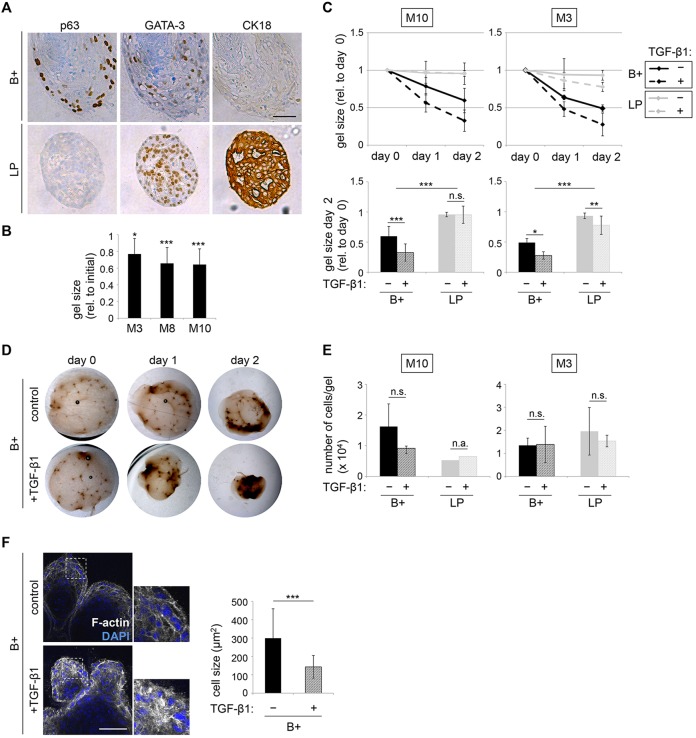
**TDLU-like structures derived from B+ cells recapitulate functional aspects of the mammary gland.** (A) Immunohistochemistry: p63, GATA3 and CK18 in representative sections of structures derived from LP or B+ cells (donor M10), fixed at culture day 20. For LP and B+, six and five fields of view were analyzed, respectively. Scale bar: 50 µm. (B) Quantification: gel size at day 13 (M3), day 14 (M8) and day 15 (M10) of culture as a percentage of the size at day 0. *n*=6 gels (M3, M10) and *n*=9 gels (M8). (C) Gel contraction: size of gels containing LP or B+ cells (donors M3, M10) at day 12 of culture (indicated as day 0) imaged for 2 days. Gel size plotted relative to day 0. Treatment with 2.0 ng/ml of TGFβ1 at day 0. Lower panel: bar graphs of gel size at day 2 as a percentage of day 0. *n*=12 gels/condition. (D) Bright-field microscopy: representative images of control and TGFβ1-treated gels containing B+ cells from C. (E) Average number of cells/gel at the end of analysis shown in C. Gels containing LP cells (donor M10) were pooled and counted. (F) Contraction of individual cells. Confocal microscopy (left): B+ cell derived structures (donor M8) were treated with TGFβ1 as in C, and stained with Phalloidin for F-actin (white) and DAPI (blue). Areas outlined are shown at higher magnification on the right. Scale bar: 100 µm. Cell size was determined for 30 cells of three different structures/condition using ImageJ area tool. n.s., not significant; n.a., not applicable. (C,E,F) Data are mean±s.d.

Therefore, we sorted B+ and LP populations of freshly isolated HMECs, plated the cells into floating collagen gels, and cultured them for a period of 20 days, to allow for differentiation. Next, immunohistochemistry was performed on paraffin-embedded sections for nuclear expression of the transcription factors p63 and GATA3, crucial determinants of basal and luminal cell fate, respectively (Asselin-Labat et al., 2007; Kouros-Mehr et al., 2006). We observed that all TDLU-like structures derived from B+ cells contained p63-positive cells in basal positions and were also GATA3-positive in luminal positions, suggesting that B+ cells gave rise to cells expressing markers of the luminal lineage (Fig. 6A). However, expression of the luminal marker cytokeratin (CK) 18 was not observed in these analyses, suggesting that CK18 might generally be induced later in the differentiation process. As expected, spheres derived from LP cells were p63 negative, but GATA3 and CK18 positive (Fig. 6A). In conclusion, our data suggest that B+ cells exhibit bipotent features in floating collagen gels by giving rise to GATA3-positive cells.

### TDLU-like structures derived from B+ cells recapitulate functional aspects of the mammary gland

A major function of the basal/myoepithelial cells in the MG is contraction of the ducts during lactation, supporting milk ejection. Indeed, we observed that gels containing TDLU-like structures began to contract after ∼12 days of culture, thus shrinking in diameter (Fig. 6B). To determine which cells exerted contractility, sorted B+ and LP cells were cultured in floating collagen gels for 12 days to allow for generation of TDLU-like structures and spheres, respectively. Gels were photographed from this time point onwards every 24 h for 2 more days. At this point, gels containing B+ cells were contracted to about one half of their initial size (Fig. 6C,D). These observations suggest that TDLU-like structures exert contractile activity in floating collagen gels, whereas spheres do not.

The morphogen TGFβ1 promotes contractility (Scharenberg et al., 2014). Indeed, a single treatment with 2.0 ng/ml recombinant TGFβ1 increased contraction of the gels containing TDLU-like structures by approximately twofold (Fig. 6C,D). By contrast, TGFβ1 did not have an effect on the size of gels containing spheres, which is in accordance with the non-contractile function of these cells *in situ*. Importantly, determining the average number of cells per gel revealed that contraction was not correlated with differences in proliferation (Fig. 6E). To confirm the increase in contraction after TGFβ1 treatment at the cellular level, detection of F-actin was performed using phalloidin, and the average cell size was determined. In accordance with the decreased gel size, single cells were significantly smaller in diameter in TGFβ1-treated structures when compared with controls (Fig. 6F).

In conclusion, contractility, an essential function of myoepithelial cells in the adult MG, is recapitulated in floating collagen gels and can be further stimulated by TGFβ1-treatment. Indeed, it was recently shown that murine MaSCs are myoepithelial and, thus, contractile (Prater et al., 2014). Therefore, determining contractility in floating collagen gels might serve as a functional assay for the identification and characterization of human MaSCs.

### Matrix compliance in floating collagen gels is necessary for alveologenesis and luminal differentiation

**Fig. 7.**
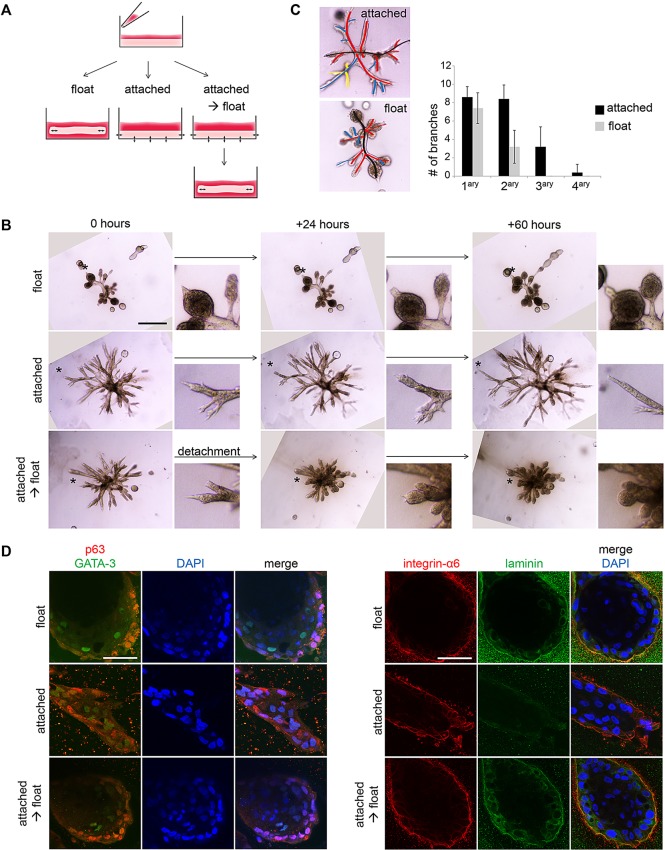
**Matrix compliance in floating collagen gels is required for alveologenesis and luminal differentiation.** (A) Experimental layout: freshly isolated HMECs were seeded into collagen I gels, which were immediately detached to float (left) or left attached to the cell culture dish (middle, right). Once branched structures had formed, some attached gels were detached (right). (B) Bright-field microscopy: representative images of HMEC-derived branched structures (donor M8), cultured according to A and imaged for 60 h, starting at day 13 of culture. Smaller images: detail of area indicated by asterisk. Scale bar: 500 µm. (C) Quantification of side branches: representative image with primary, secondary and tertiary side branches indicated by red, blue and yellow lines, respectively. Graph shows the number of side branches/structure in attached and floating collagen gels at day 13 of culture for five structures/condition (donor M8). Data are mean±s.d. (D) Confocal microscopy: representative images of HMEC-derived branched structures (donor M8), cultured according to A,B: p63 (red), GATA3 (green), integrin α6 (red), laminin (green) and DAPI for nuclei (blue). Scale bars: 50 µm.

To test whether contraction of gels is required for formation of TDLU-like structures, HMECs were cultured either in floating collagen gels or in gels that remained attached to the bottom and walls of the polystyrene culture dish, thereby preventing gel contraction. Additionally, HMECs were plated into attached collagen gels that were detached to float, once branched structures had formed (Fig. 7A). Substantial differences in morphology were displayed: while cells in floating gels developed alveoli at the tips of branched structures, cells in attached gels formed thin and elongated ducts with a significantly increased number of side branches and complete lack of alveologenesis (Fig. 7B,C). Remarkably, formation of alveoli could be induced within 24 h in attached gels that were detached to float (Fig. 7B). Together, these results indicate that a rigid collagen matrix that cannot be contracted by B+ cells promotes elongation and side branching, whereas a compliant matrix in floating gels promotes alveologenesis.

To further investigate whether switching from a rigid to a compliant matrix environment promoted differentiation at the cellular level, we performed confocal immunofluorescence. In floating/compliant collagen gels, cells of the outer layers adjacent to the collagen matrix expressed the basal marker p63. By contrast, cells in the inner layer were p63 negative and expressed the transcription factor GATA3 and the tight-junction protein ZO-1 at luminal positions (Fig. 7D; supplementary material Fig. S4A), consistent with our earlier observations (Fig. 6A) and similar to lineage marker expression *in situ*. Furthermore, integrin α6 (CD49f) was exclusively localized at the basal position and colocalized with its ligand laminin, indicating deposition of basement membrane components by the basal cell layer. By contrast, branched structures in attached/rigid gels did not form round buds and showed no polarized expression of p63 and integrin α6, and low to undetectable levels of laminin (Fig. 7B,D). Furthermore, ZO-1 was not detectable in branched structures within attached collagen gels, whereas GATA3 staining was observed only in rare cells that were localized at both basal and luminal positions (Fig. 7D; supplementary material Fig. S4A). These observations were further supported by the finding that mRNA levels of *ELF5* and *TJP1*/ZO-1 were not detectable or lower in B+ cell-derived branched structures grown in attached compared with floating collagen gels (supplementary material Fig. S4B). However, expression of *GATA3* mRNA was detected in all conditions (data not shown). As expected by the non-contractile function of luminal cells, attachment of the gels did not have any detectable effect on the morphology, as well as on the expression of *ELF5* and *TJP1*/ZO-1 in LP-derived spheres (supplementary material Fig. S4B,C). Taken together, these results indicated that culture within a floating/compliant collagen matrix promotes alveologenesis and luminal differentiation of basal HMECs.

To test whether contractility of basal cells was required for alveologenesis, we again plated freshly isolated HMECs into attached collagen gels. Once branched structures had formed, gels were detached and simultaneously treated with either the myosin II inhibitor blebbistatin (Prater et al., 2014) or the ROCK inhibitor Y-27632 to prevent cellular contraction (supplementary material Fig. S4D,E). Although structures in the control condition acquired an alveolar morphology after detachment, this was prevented by treatment with either of the compounds (supplementary material Fig. S4F). Together, these results indicate that the contractile function of basal cells is crucial for alveologenesis and differentiation.

## DISCUSSION

When cultured in collagen I gels using defined conditions, freshly isolated basal HMECs give rise to complex branched multicellular structures. However, we observed the generation of alveoli-like buds and differentiation towards the luminal lineage only in floating/compliant, but not in attached/rigid, collagen gels. Consistently, previous studies demonstrated that matrix rigidity regulates growth and morphogenesis of breast epithelial cells in 3D-culture models. Specifically, high stiffness due to collagen concentration, crosslinking, matrix attachment or interpenetrating polymer networks has been shown to disrupt normal morphogenesis, leading to multicellular structures with a malignant/invasive phenotype (Chaudhuri et al., 2014; Levental et al., 2009; Paszek et al., 2005; Provenzano and Keely, 2009; Wozniak et al., 2003). Indeed, we observed persistent elongation and increased formation of side branches by primary HMECs in rigid collagen gels, a process that inherently requires invasiveness and was related to a basal epithelial program in invasive breast cancer, also using collagen gels as an experimental tool (Cheung et al., 2013). While high matrix stiffness is observed in breast tumors and has been linked to tumor progression (Levental et al., 2009; Paszek et al., 2005), our results suggest that responsiveness to the physical environment is a conserved feature in normal MG development. Notably, it has been suggested that tensional homeostasis of both exogenous and endogenous forces, mediated via the cytoskeleton, is crucial for normal tissue behavior (Paszek and Weaver, 2010). Such endogenous forces may be particularly important, as MaSCs display contractility (Prater et al., 2014).

Although flotation of the gels was required for alveologenesis at the tips of branched structures, addition of forskolin, which raises intracellular cAMP levels, increased the generation of alveoli in floating gels. As cAMP accelerates the distribution of integrin α6 to the periphery (Nedvetsky et al., 2012), forskolin probably enhanced cell adhesion to laminins. Importantly, we observed laminin staining only in structures grown in floating, but not attached, collagen gels. Together, these results suggest that the effects of both flotation and forskolin impinge on anchoring epithelial cells to the basement membrane as a prerequisite to establish basal polarity, thereby promoting alveologenesis.

Primary cells most accurately reflect cell behavior *in vivo*, and supposedly differences between donors, including age, parity, hormonal status and genetic background (Tanos et al., 2013). Therefore, heterogeneity in TDLU-like structure-forming potential among donor tissues was expected and suggests that individual differences are conserved in culture. For our limited number of tissues, TDLU-like structure formation did not correlate with age or parity of the donors, suggesting that cells with regenerative potential are maintained even after menopause and exist before pregnancy.

Importantly, the use of the metallo-endopeptidase CD10 as a cell-surface marker revealed the presence of a CD10-negative stromal component within the CD49f^hi^/EpCAM^−^ population, previously labeled basal. Gene expression profiling suggested that this stromal component may contain a variety of CD31^−^ and CD45^−^ endothelial and hematopoietic cells. Given the relevance of interactions between HMECs and lymphendothelium during branching morphogenesis (Betterman et al., 2012) and breast cancer progression (Alitalo and Detmar, 2012), future work will focus on the characterization of this distinct stromal component. For this purpose, the chemically and physically defined *in vitro* assay system we describe here will be particularly useful: stromal components can be added for co-culture studies and HMECs with distinct genetic backgrounds can be tested.

Luminal progenitor cells of two out of three donors gave rise to branched structures, suggesting that plasticity can occur in this compartment. Future studies will address whether plasticity in the luminal compartment reflects normal regeneration or a process of illicit dedifferentiation, as was suggested to occur in *BRCA1*-mutant luminal progenitors (Lim et al., 2009). Importantly, rigorous quantification of normal or malignant regenerative capacity at the single-cell level is enabled by ELDA. Finally, for future studies, it will be important to test whether our assay can be adapted to support cells of other organs undergoing branching morphogenesis, such as lung, kidney or pancreas (Lu et al., 2006).

## MATERIALS AND METHODS

### Isolation and culture of human mammary epithelial cells

Mammary gland tissue was obtained from healthy women undergoing reduction mammoplasty at the Nymphenburg Clinic for Plastic and Aesthetic Surgery, in accordance with the regulations of the ethics committee of the Ludwig-Maximilian University, Munich, Germany (proposal 397-12). Single-cell suspensions of primary HMECs were generated as previously described with minor modifications (Stingl et al., 2005). Briefly, the ductal tree was minced into about 1.0 mm^3^ pieces and digested sequentially using collagenase I together with hyaluronidase (both Sigma), Trypsin-EDTA and dispase (Life Technologies), and then cryopreserved. Before further processing, cells were filtered through a 40 µm strainer, to remove tissue fragments and cell aggregates. Cells were seeded in 2D on polystyrene cell culture plates or in collagen I gels in mammary epithelial cell growth medium (MECGM, PromoCell) supplemented with 1% pen/strep (Invitrogen), 0.5% FCS (Pan Biotech), 3 µM Y-27632 (Biomol) and 10 µM forskolin (Biomol), unless stated otherwise. After an establishment period of 5 days, medium was changed to MECGM supplemented with 1% pen/strep and 10 µM forskolin, unless stated otherwise.

### 3D collagen I gels

Single-cell suspensions were quickly mixed with neutralizing solution, and acidified rat tail collagen I (Corning) was added, resulting in a final concentration of 1.3 mg/ml. Next, the gel mixture was plated into siloxane-coated 24-well or 48-well plates. After polymerization of the gel, medium with supplements was added and gels were detached from the well. Attached gels were prepared in uncoated 24-well plates.

### Extreme limiting dilution analysis (ELDA)

For determination of structure-forming units (SFU), at least six gels per cell dose were prepared in 48-well plates, as described above. Structures were stained with Carmine solution and imaged on a Zeiss SteREO Lumar.V12 microscope with a NeoLumar S 0.8× objective (10-20× zoom). Gels with at least one branched structure were counted as positive. Branched structures were defined as containing at least two branching points and being 0.057 mm^2^ or more in size. Limiting dilutions were analyzed as described previously (Hu and Smyth, 2009).

### Immunofluorescence

Cells were fixed with 4% paraformaldehyde. For immunofluorescence, cells were permeabilized with 0.2% Triton X-100 and blocked with 10% goat or donkey serum in 0.1% BSA. Primary and secondary antibodies used for stainings are listed in supplementary material Tables S3 and S4. Cell nuclei were visualized with DAPI.

### Flow cytometry and fluorescence-activated cell sorting (FACS)

Single-cell suspensions of HMECs were stained with CD31-PB, CD45-V450, CD49f-PE, EpCAM-FITC and CD10-APC antibodies (supplementary material Table S5). Prior to sorting, 7AAD (BD Biosciences) was added to distinguish dead and live cells. After excluding 7AAD^+^ and CD31^+^/CD45^+^ (Lin^+^) cells, HMECs were sorted into three or four populations (LP: CD49f^+^/EpCAM^+^, B: CD49f^hi^/EpCAM^−^, B−: CD10^−^/CD49f^hi^/EpCAM^−^ and B+: CD10^+^/CD49f^hi^/EpCAM^−^) using a FACS Aria III (BD Biosciences). The separated populations were re-analyzed to ensure the purity of the sort. FlowJo V10 was used for post-analysis.

### Expression profiling and statistical transcriptome analysis

Total RNA from freshly sorted HMECs from donors M3, M6, M8, M9, M10 and M12 was amplified using the Ovation Pico WTA System V2 in combination with the Encore Biotin Module (Nugen). Amplified cDNA was hybridized on Affymetrix Human Gene 2.0 ST arrays. Array data have been submitted to GEO (Accession Number GSE64248).

### Statistical analysis

Data are presented as mean±s.d. except for SFUs, which are shown as mean and 95% confidence intervals (CI). Student's *t*-test (two-tailed, unpaired) was used to compare two groups. *P*<0.05 was considered significant: *P<0.05, **P<0.005, ***P<0.0005.

### Expanded materials and methods

See supplementary material for expanded materials and methods, including primer sequences (Table S2).

## Supplementary Material

Supplementary information
