# Supplementary material for: Quantification of regenerative potential in primary human mammary epithelial cells
Source: Development. 2015 Sep 15;142(18):3239–51. doi: 10.1242/dev.123554 (PMC4582177; doi:10.1242/dev.123554)
Supplement: Supplementary information [file supp_142_18_3239__index.html]

Supplementary information 

# Quantification of regenerative potential in primary human mammary epithelial cells

## DEV123554 Supplementary information

- Supplementary information
