# Supplementary material for: Quantification of regenerative potential in primary human mammary epithelial cells
Source: Development. 2015 Sep 15;142(18):3239–51. doi: 10.1242/dev.123554 (PMC4582177; doi:10.1242/dev.123554)
Supplement: Supplementary information [file supp_dev.123554_DEV123554supp.pdf]

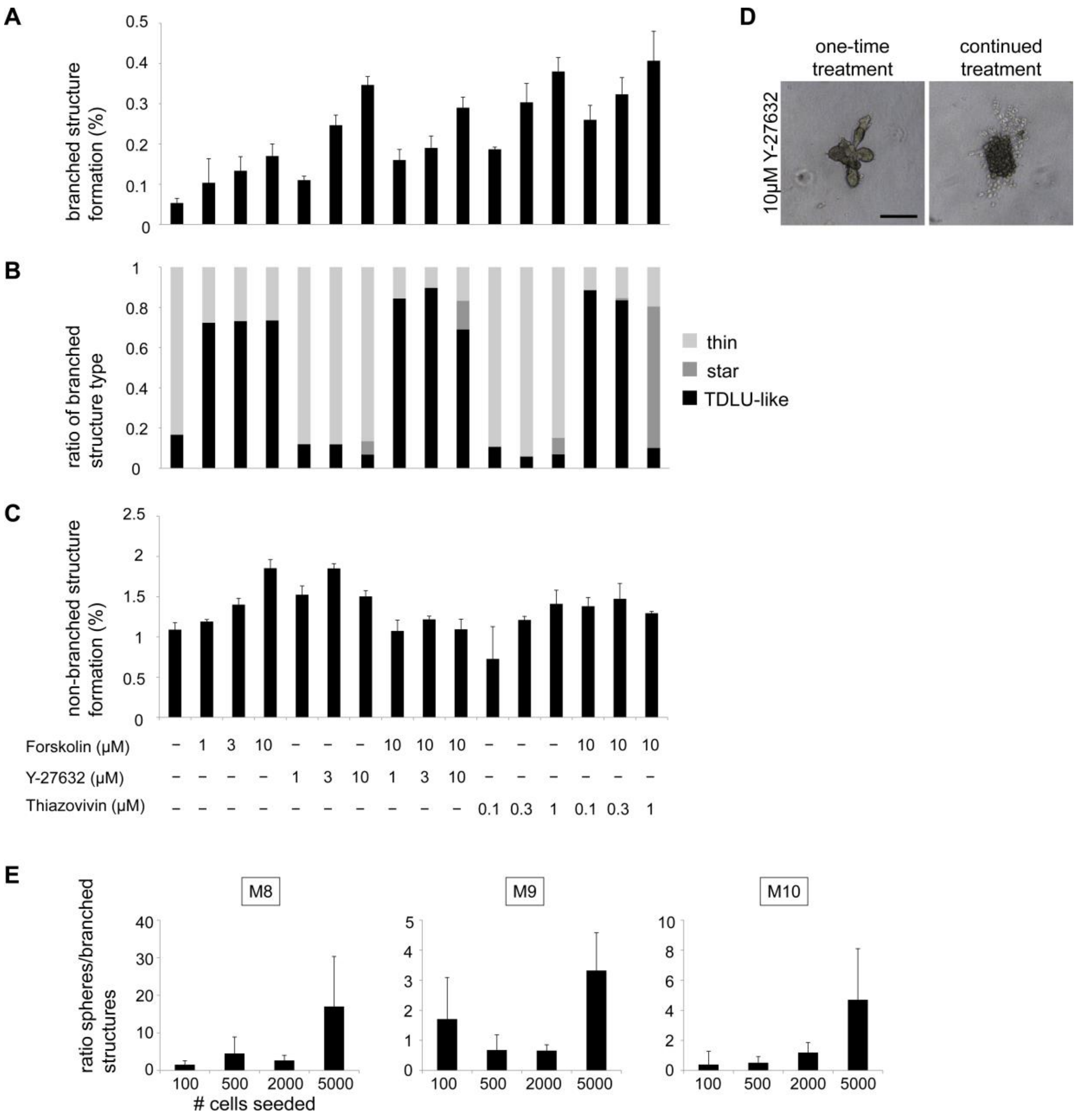

Figure S1 referring to Figure 1. Identification of culture conditions that promote generation of TDLU-like structures by freshly dissociated HMECs

(A) Effect of culture conditions on the generation of branched structures: HMECs (donor M8) were cultured in presence of different concentrations of Forskolin (continuous treatment), Y-27632 and Thiazovivin (both one-time treatment at day

Development • Supplementary information

0 of culture) in floating collagen gels for 14 days. n=3 gels/condition. Structure formation is given per 100 seeded cells.

(B) Effect of culture conditions on the ratio of branched structure subtypes, refer to (A). n=3 gels/condition.

(C) Effect of culture conditions on the generation of non-branched structures, refer to (A). n=3 gels/condition. Structure formation is given per 100 seeded cells.

(D) Bright-field microscopy: representative images of M5 basal cells in floating collagen gels, treated with 10 μM Y-27632 one-time at day 0 of culture or treated with every medium change. Scale bar: 200 µm.

(E) Quantification of the ratio of spheres and branched structures in floating collagen gels (used for analysis of clonality in Fig. 1E) containing 100, 500, 2000 and 5000 cells/well (24-well plate). n=5 fields of view of at least 6 gels/condition.

Data are shown as mean ± standard deviation (SD).

Development • Supplementary information

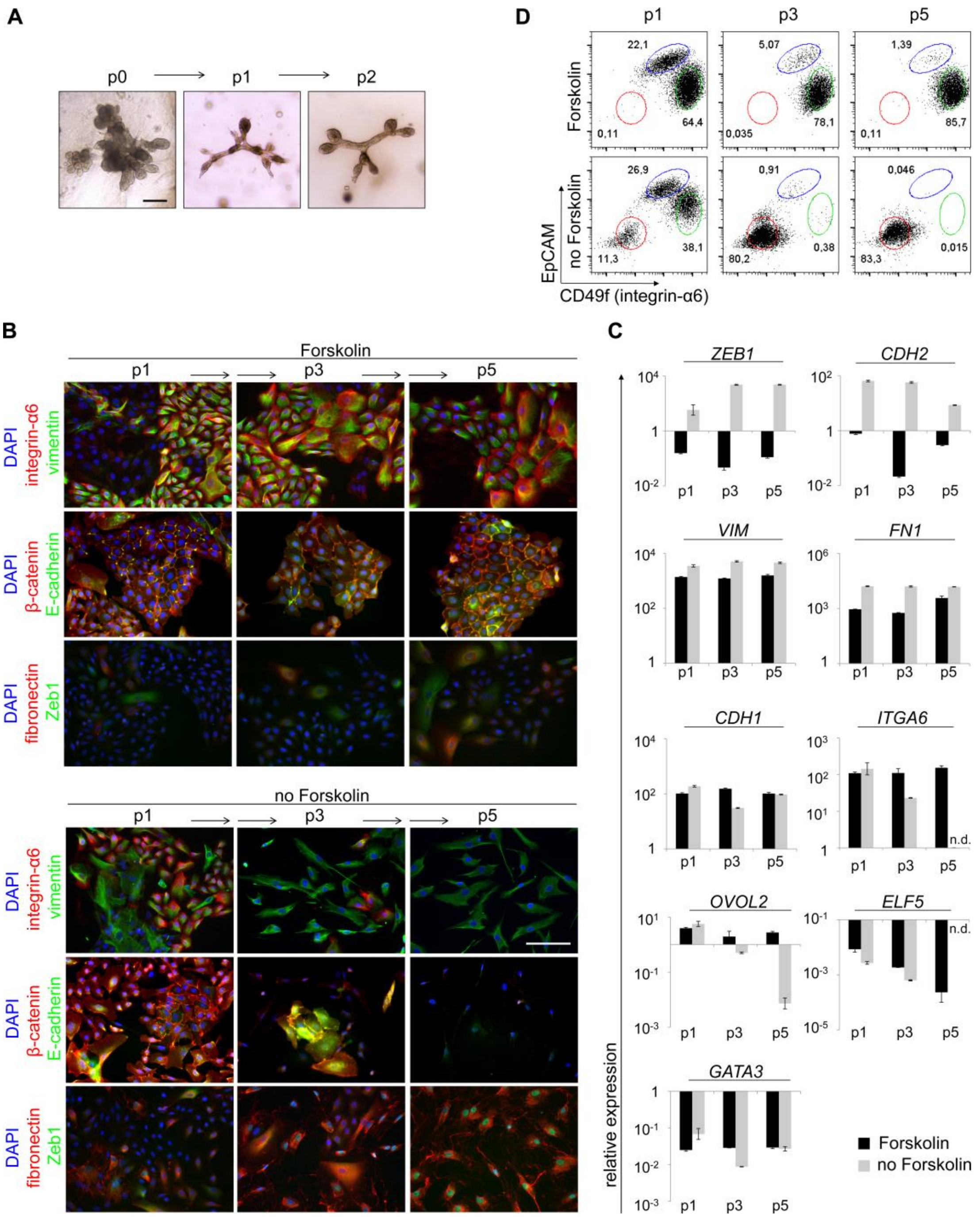

Figure S2 referring to Figure 2. Maintenance and expansion of TDLU-like structure formation during passaging and 2D-culture

(A) Bright-field: representative images of HMEC-derived branched structures (donor M8), at subsequent passages in 3D. Scale bar: 200 µm.

Development • Supplementary information

(B) 2D-Immunofluorescence: representative images of HMECs cultured in 2D, in the absence or presence of 10 µM Forskolin at passage 1, 3 and 5 (donor M4). Integrin-α6 (red), vimentin (green), β-catenin (red), E-cadherin (green), fibronectin (red), Zeb1 (green), DAPI (blue). Scale bar: 100 µm.

(C) RT-PCR: *ZEB1*, *CDH2* (N-cadherin),*VIM* (vimentin), *FN1* (fibronectin) and *CDH1* (E-cadherin), *OVOL2*, *ITGA6* (integrin-α6), *ELF5* and *GATA3* mRNA expression of HMECs cultured in 2D, as described in (B). n=3.

(D) Flow cytometry analysis of CD49f and EpCAM expression in Lin^- HMECs cultured in 2D, as described in (B).

p, passage.

n.d., not detectable

Development • Supplementary information

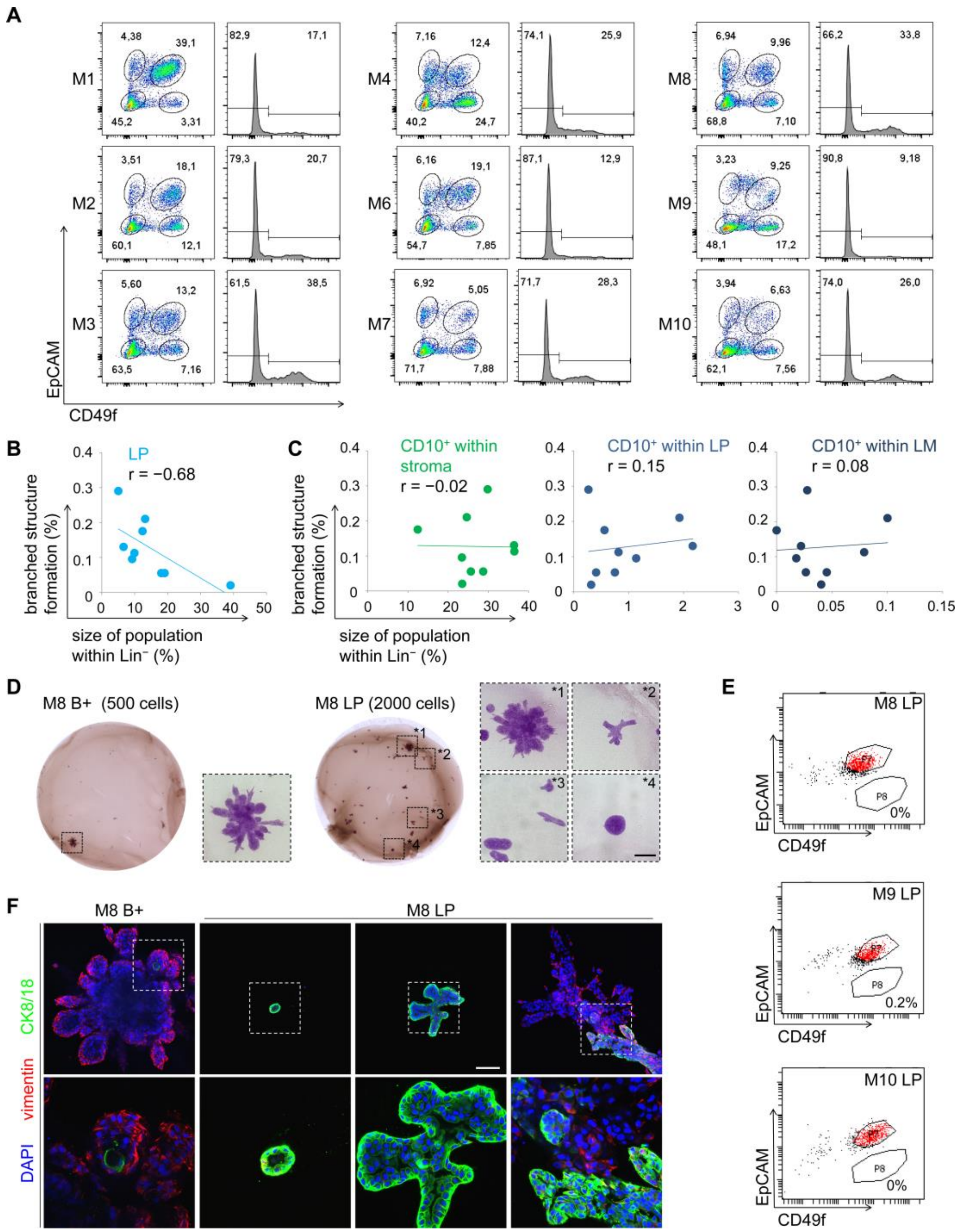

Figure S3 referring to Figure 4. TDLU-like structure-forming potential is contained within a CD10⁺/CD49f^{hi}/EpCAM⁻ basal population

(A) Flow cytometry analysis of CD49f, EpCAM and CD10 expression in the 7-AAD⁻, Lin⁻ subset of freshly isolated HMECs from 9 donors (M1-M4, M6-M10) used in

Development • Supplementary information

Figs 3, 4. Determined population sizes were used for correlation analysis in Figs 4B,C, S3B,C.

(B) Correlation between branched structure formation and the size of the LP population. One dot represents one donor.

(C) Correlation between branched structure formation and the size of the CD10+ stromal population ($CD10^{+}/CD49f^{-}/EpCAM^{-}$, green dots), the $CD10^{+}$ LP population ($CD10^{+}/CD49f^{+}/EpCAM^{+}$, blue dots), and CD10+ LM population ($CD10^{+}/CD49f^{-}/EpCAM^{+}$, dark blue dots). One dot represents one donor.

(D) Bright-field: representative images of structures derived from LP and B+ sorted cells (donor M8) from ELDA experiment in Fig. 4D. Scale bar: 200 µm.

(E) Reanalysis of the purity of sorted LP cells from donors M8, M9 and M10 used for ELDA in Fig. 4E.

(F) Confocal microscopy: representative images of structures derived from LP and B+ sorted cells (donor M8). CK8/18 (green), vimentin (red), DAPI (blue). Scale bar: 100 µm.

r, correlation coefficient.

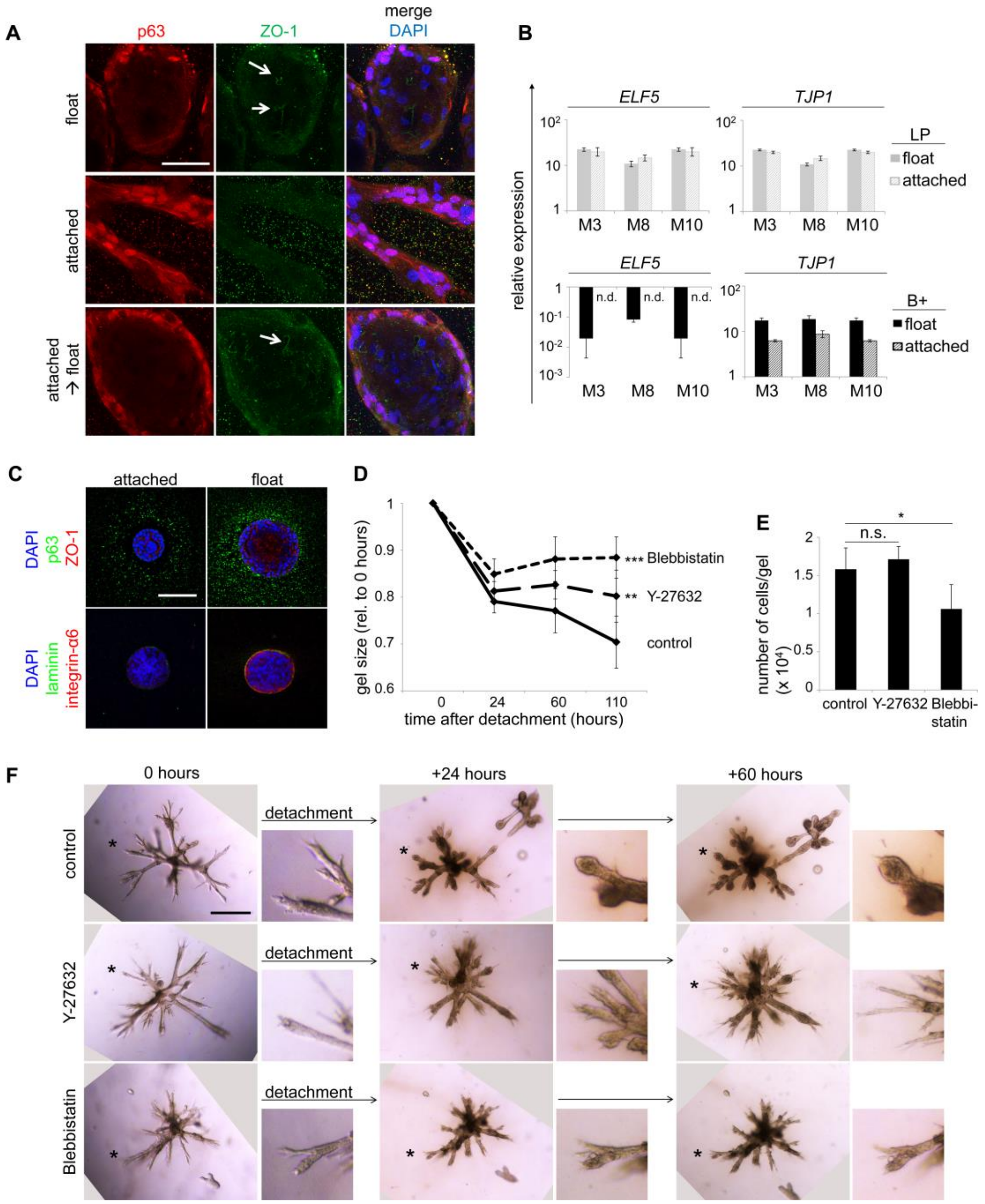

Figure S4 referring to Figure 7. Matrix compliance in floating collagen gels is necessary for alveologenesis and luminal differentiation of TDLU-like structures.

(A) Confocal microscopy: representative images of HMEC-derived branched structures (donor M8), cultured according to Fig. 7A,B. p63 (red), ZO-1 (green), DAPI (blue). Arrows point to ZO-1 expression. Scale bar: 50 µm.

Development • Supplementary information

(B) RT-PCR: *ELF5* and *TJP1* (ZO-1) mRNA expression in B+ and LP cell derived structures from donors M3, M8 and M10, cultured in attached and floating collagen gels. n=3.

(C) Confocal microscopy: representative images of HMEC-derived spheres (donor M8), cultured in floating and attached collagen gels, at day 14 of culture. p63 (green), ZO-1 (red), integrin-α6 (red), laminin (green), DAPI (blue). Scale bar: 100 µm.

(D) Contraction of collagen gels: HMECs from donor M10 were grown in attached collagen gels. Once branched structures had formed, gels were detached (day 13 of culture) and treated with 10 µM Blebbistatin or 5 µM Y-27632 every 24 hours. The size of the gels was determined directly after detachment (0 hours), and after 24, 60 and 110 hours. Gel size is plotted relative to the time point of detachment (0 hours). n=16 gels/condition.

(E) Quantification of the average number of cells per gel at the end of analysis shown in (D), n=4.

(F) Bright-field microscopy: representative images of HMEC-derived branched structures (donor M10) cultured in attached collagen gels for 12 days, detached on day 13 of culture, and treated with 10 µM Blebbistatin or 5 µM Y-27632 every 24 hours. Structures were imaged for 60 hours. Smaller pictures are details of areas indicated with asterisk. Scale bar: 500 µm.

n.d., not detectable

n.s., not significant

Development • Supplementary information

SUPPLEMENTARY TABLES

Table S1. Reduction mammoplasty donors

Donor	Age (years)	Parity
M1	44	1
M2	68	1
M3	71	2
M4	68	2
M6	69	1
M7	35	2
M8	53	2
M9	17	0
M10	42	1
M12	54	0

Table S2. Primers used for qPCR

Target	Sequence (Fw, Rv)
CDH1	TGCCCAGAAAATGAAAAAGG, GTGTATGTGGCAATGCGTTC
ELF5	TAGGGAACAAGGAATTTTTCGGG, GTACACTAACCTTCGGTCAACC
FN1	CAGTGGGAGACCTCGAGAAG, TCCCTCGGAACATCAGAAAC
GATA3	GCCCCTCATTAAGCCCAAG, TTGTGGTGGTCTGACAGTTCG
KRT8	TCCTCAGGCAGCTATATGAAGAG, GGTTGGCAATATCCTCGTACTGT
RPL32	CAGGGTTCGTAGAAGATTCAAGGG,CTTGGAGGAAACATTGTGAGCGATC
MME	TGGATCTTGTAAGCAGCCTCA, GCACAACGTCTCCAAGTTGC
CDH2	ACAGTGGCCACCTACAAAGG, CCGAGATGGGGTTGATAATG
OVOL2	ACAGGCATTCGTCCCTACAAA, CGCTGCTTATAGGCATACTGC
TP63	AGAGAGAGGGACTTGAGTTCT, TGGTCGATGCTGTTCAGGAGC
SNAI2	GGGGAGAAGCCTTTTTCTTG, TCCTCATGTTTGTGCAGGAG
VIM	GAGAACTTTGCCGTTGAAGC, GCTTCCTGTAGGTGGCAATC
ZEB1	GCACAAGAAGAGCCACAAGTAG, GCAAGACAAGTTCAAGGGTTC
TJP1	CTTACCACACTGTGCGTCCAT, AGGAGTCGGATGATTTTAGAGCA

Development • Supplementary information

Table S3. Primary antibodies for immunohistochemistry and immunofluorescence

Immunohistochemistry

Epitope [Clone]	Conjugation	Host	Supplier
GATA3 [L50-823]	-	mouse	Biocare Medical (CM405)
CK18 [Ks18.04]	-	mouse	Progen (61028)
p63 [BC4A4]	-	mouse	Biocare Medical (PM163AAK)

Immunofluorescence

Epitope [Clone]	Conjugation	Host	Supplier
K8/18 [5D3]	-	mouse	Dianova, Hamburg
E-cadherin [24E10]	Alexa 488	rabbit	NEB, Whitby, Canada
E-cadherin [EP700Y]	-	rabbit	Biozol, Eching
GATA-3 [L50-823]	-	mouse	Biocare Medical (CM405)
integrin-α6 [GOH3]	-	rat	Santa Cruz, Dallas, USA
laminin	-	rabbit	Sigma, Steinheim

p63 [BC4A4]	-	mouse	Abcam, Cambridge, UK
p63 [H-137]	-	rabbit	Santa Cruz, Dallas, USA
Phalloidin	Atto 647N	-	Sigma, Steinheim
vimentin [D21H3] XP	-	rabbit	Biozol, Eching
vimentin [V9]	-	mouse	Abcam, Cambridge, UK
ZO-1	Alexa 594	mouse	Invitrogen, Karlsruhe
ZO-1 [1A12]	-	mouse	Life Technologies

Table S4. Secondary antibodies

Host	Epitope	Conjugation	Supplier
Goat	Mouse IgG	Alexa 594	Life Technologies, Darmstadt
Goat	Rabbit IgG	Alexa 488	Life Technologies, Darmstadt
Donkey	Mouse IgG	Alexa 488	Life Technologies, Darmstadt
Donkey	Rabbit IgG	Alexa 546	Life Technologies, Darmstadt
Donkey	Rabbit IgG	Alexa 488	Life Technologies, Darmstadt
Donkey	Rabbit IgG	Alexa 594	Life Technologies, Darmstadt
Donkey	Rat IgG	Cy3	Dianova, Hamburg

Development • Supplementary information

Table S5. Antibodies used for flow cytometry and fluorescence activated cell sorting

Epitope [Clone]	Conjugation	Host	Supplier
7-AAD	-	-	BD, Heidelberg
CD10 [HIC10a]	APC	mouse	Biozol, Eching
CD31 [WM59]	PB	mouse	Biozol, Eching
CD326/EpCAM [VU-1D9]	FITC	mouse	Biozol, Eching
CD45 (HI30]	V450	mouse	BD, Heidelberg
CD49f [GoH3]	PE	rat	BD, Heidelberg

Development • Supplementary information

SUPPLEMENTARY MATERIALS AND METHODS

Expanded procedure: isolation and culture of human mammary epithelial cells

Mammary gland tissue was obtained from healthy women undergoing reduction mammoplasty at the Nymphenburg Clinic for Plastic and Aesthetic Surgery (Prof. Christian Gabka), in accordance with the regulations of the ethics committee of the Ludwig-Maximilian University Munich (proposal 397-12). Single cell suspensions of primary HMECs were generated as previously described with minor modifications (Stingl et al., 2005). Briefly, the ductal tree was minced into about 1 mm^3 pieces and enzymatically digested in tissue digestion buffer (F12:DME/HEPES, 1,5% w/v BSA) supplemented with 1 µg/ml insulin, 300 U/ml collagenase and 100 U/ml hyaluronidase (all Sigma) at 37°C over night. The stromal compartment was optionally separated by differential centrifugation and cryopreserved. The pellet enriched for epithelial cells was further dissociated in 0.15% Trypsin-EDTA and 5 mg/ml dispase (Life Technologies) and then cryopreserved. Before further processing, cells were filtered through a 40 µm strainer, to remove residual tissue fragments and cell aggregates. Freshly isolated primary HMECs were seeded in Mammary Epithelial Cell Growth Medium (MECGM, PromoCell) supplemented with 1% Pen/Strep (Invitrogen), 0.5% FCS (Pan Biotech), 3 µM Y-27632 (Biomol) and 10 µM Forskolin (Biomol), unless otherwise stated. After an establishment period of 5 days, medium was changed to MECGM supplemented 1% Pen/Strep and 10 µM Forskolin, unless otherwise stated. Upon establishment, medium was replaced every 3-4 days. Cells were maintained in 5% CO_2, 3% O_2 for the whole culture period.

Expanded procedure: 3D-collagen gels

In case of floating collagen gels tissue culture plastics were siloxane-coated by pretreatment with a solution of 25 g/l dichloro-octamethyltetrasiloxane (Santa Cruz, sc-229834) in n-heptane (Applichem, #1948) for approximately 30 seconds and subsequently rinsed one time each with PBS and water. Siloxane-coating facilitates detachment of gels. For attached or attached-to-floating collagen gels the culture plates were left uncoated.

Three-dimensional floating collagen gels were prepared based on a published protocol (Wozniak and Keely, 2005) with modifications described below.

Neutralizing solution (11x PBS, 550 mM HEPES, comprising 1/10th of the volume of collagen) was added to a single cell suspension in growth medium containing the desired amount of cells. Quickly, acidified rat tail collagen type I (Corning) was added, resulting in a final concentration of collagen of 1.3 mg/ml. Next, the gel mixture was quickly plated into 24-well (400 µl) or 48-well (200 µl) tissue culture plastics on ice and left to polymerize at 37°C for 1 hour after which 600 µl (24-well plate) or 300 µl (48-well plate) medium with supplements was carefully added. The concentrations of supplements were calculated for the total volume of the gel with medium.

In case of floating collagen gels, the gels were detached from the well by encircling them with a pipet tip followed by gently shaking the plate. Cells were cultured for 8 up to 20 days.

For improvement of culture conditions, 1x10^4 HMECs/400 µl collagen gel were seeded. For comparison of structure formation by 9 different donors in passage 0 and in

Development • Supplementary information

passage 2, $2x10^4$ HMECs/400 µl collagen gel and $8x10^2$ HMECs/400 µl collagen gel were plated, respectively.

For contraction assays, $5x10^3$ HMECs were plated or $3x10^3$ sorted B+ cells/400 µl collagen gel and $1x10^4$ LP cells/400 µl collagen gel. At day 12 of culture, 2 ng/µl TGF-β1 (R&D Systems) was added to the culture medium once. For inhibition of contraction experiments, $3x10^3$ HMECs (donor M10) were plated/400 µl collagen gel, and the gels were left attached to the culture dish. At day 13 of culture, when structures had formed, gels were detached and 10 µM Blebbistatin or 5 µM Y-27632 were added to the culture medium every 24 hours. To determine the number of cells per gel, collagen gels were minced using a scalpel, digested with 300 U/ml collagenase I (Sigma) for 1 hour at 37ºC, followed by 0.15% trypsin (5 minutes at 37ºC), and filtered to obtain single cells. Cells were counted with a hemocytometer. Images of structures in the gels were acquired on a Leica DM IL LED microscope equipped with a HiPlan 10x/0.22 PH1 objective and images of whole gels were taken with a Zeiss SteREO Lumar.V12 microscope with a NeoLumar S 0.8x objective (6.4 x Zoom).

3D-Matrigel culture

Single cells were resuspended in Growth Factor Reduced Matrigel (Corning), plated into 24-well plates on ice (400 µl/well) and Matrigel was left to polymerize at 37°C for 1 hour. After this, medium was added and gels were treated like the 3D-collagen gels.

Development • Supplementary information

Expanded procedure: 2D-immunofluorescence

Cells grown on poly-D-lysine-coated glass coverslips were fixed with 4% paraformaldehyde for 15 minutes, permeabilized with 0.2% Triton X-100 for 2 minutes, and then blocked with 10% goat or donkey serum in 0.1% BSA for 1 hour. Slides were incubated with primary antibodies in 0.1% BSA for 1 hour, followed by incubation with secondary antibodies in 0.1% BSA for 2-3 hours. Cell nuclei were stained with 167 ng/ml DAPI. Coverslips were mounted with AQUA-POLY/MOUNT mounting medium (Polysciences). All steps were performed at room temperature. Images were acquired on an Axioplan 2 imaging light/fluorescence microscope using a 20x objective and processed with Axiovision Rel 4.7 and Gimp 2.8.2/Adobe Photoshop CS5 software.

Expanded procedure: 3D-immunofluorescence

Cells in 3D collagen gels were washed with PBS for 10 minutes, fixed with 4% paraformaldehyde for 15 minutes, washed with PBS for 10 minutes, quenched with 0.15 M Glycine for 10 minutes, and washed again with PBS for 10 minutes. Then, cells were permeabilized with 0.2% Triton-X-100 for 10 minutes and washed with PBS for 10 minutes. Cells were blocked with 10% goat or donkey serum (both Biozol) in 0.1% BSA for 3 hours at room temperature or overnight at 4°C. After washing with PBS for 10 minutes, gels were incubated with primary antibodies in 0.1% BSA at 4°C overnight. Gels were washed with PBS three times for 10 minutes and incubated with secondary antibodies in 0.1% BSA for 2-3 hours at room temperature, followed by further two times washing with PBS for 10 minutes (for antibodies, see Tables S3,4). Cell nuclei were

Development • Supplementary information

stained with 167 ng/ml DAPI (Sigma) for 2 minutes. Then, gels were washed with PBS three times for 10 minutes and with water two times for 5 minutes. The fixation, quenching, permeabilization, and all washing steps were performed at room temperature on a shaker. Collagen gels were transferred to a microscope slide, excess liquid was removed with a tissue, and mounted with AQUA-POLY/MOUNT mounting medium (Polysciences). Samples were imaged on an inverted confocal laser scanning microscope equipped with 4 laser lines (405, 488, 543, and 633 nm) and UPLSAPO 60x, 40x and 20x objective lenses. FV-10-ASW 1.7 Viewer and Gimp 2.8.2/Adobe Photoshop CS5 software were used to adjust brightness across the entire image field.

Immunohistochemistry

For immunohistochemistry, collagen gels were fixed in 4% paraformaldehyde and embedded in paraffin. Staining was performed on 2 µm thick sections according to manufacturer's recommendations and standard protocols. Antibodies are listed in Table S3 and were detected with the ultraView Universal DAB Detection Kit (Roche). For hematoxylin and eosin staining, formalin-fixed and paraffin-embedded (FFPE) breast tissues from cosmetic breast reduction surgeries were selected from the tissue archives of the Institute of Pathology, Ludwig-Maximilians-University Munich, Munich, Germany. 2 µm thick H&E-stained sections were examined by two pathologists for no evidence of dysplasia or malignancy. Tissue samples had been anonymized according to the local ethics committee regulations.

Carmine staining

Carmine-alum solution was prepared according to standard protocols. Collagen gels were fixed with 4% paraformaldehyde, as described above, and were incubated in Carmine solution on a shaker overnight at room temperature and then mounted with Roti-Aqua Mount (Roth). Structures in gels were imaged on a Leica DM IL LED microscope with a HiPlan 10x/0.22 PH1 objective and whole mount pictures were taken with a Zeiss SteREO Lumar.V12 microscope with a NeoLumar S 0.8x objective (10-20x Zoom).

RNA preparation and quantitative PCR analysis

After homogenization using the QIAshredder, RNA was isolated with the RNeasy Mini Kit in combination with the RNase-Free DNase Set (all Qiagen), according to manufacturer's instructions. RNA was reverse transcribed using the EasyScript Plus cDNA Synthesis Kit (Abm) according to the manufacturer's Oligo(dT) protocol. In case of small amounts of RNA, total RNA was amplified using the Ovation Pico WTA System V2 in combination with the Encore Biotin Module (Nugen). Real-Time quantitative PCR was performed with the Power SYBR Green PCR Master Mix (Life Technologies) on a QuantStudio 12K Flex qPCR System. Data were analyzed using the ΔCt method to present data as fold change expression compared to the housekeeping gene *RPL32* (Schmittgen and Livak, 2008). Primers are listed in Table S2.

Development • Supplementary information

Morphological analysis of gels, structures and cells

Size of gels, structures, and cells was determined with the ImageJ tool for measurement of areas. Quantification of structures was carried out using the ImageJ cell counter. Structures with at least two branching points were considered as branched. For branching point analysis, branches were traced, one main branch was set, and one branching point was counted for each side-branch.

Plasmids, virus production and infection of target cells

The mCherry coding sequence was amplified using primers mCherry_XbaI_FW (ttTCTAGAcaggatcccgccaccatg) and mCherry_SalI_RV (ttGTCGACttacttgtacagctcgtccatgc) and cloned into pRRL.SIN.cPPT.CMV-GFP.WPRE (gift from Timm Schröder, ETH Basel, Switzerland) using XbaI and SalI. HEK293T high performance cells (ATCC) were transfected with pMD2.G (Addgene plasmid 12259), psPAX2 (Addgene plasmid 12260), and pRRL coding either for GPF or mCherry. Cell-free supernatants were collected during 48 hours and 1 ml of lentiviral suspension were applied per well (6-well plate) of HMECs passage 0, in the presence of 3.3 µg/ml protamine sulfate. After 4 hours, cells were trypsinized and seeded into floating collagen gels.

Expanded procedure: Expression profiling and statistical transcriptome analysis

Total RNA from freshly sorted HMECs from donors M3, M6, M8, M9, M10, M12 was amplified using the Ovation Pico WTA System V2 in combination with the Encore Biotin Module (Nugen). Amplified cDNA was hybridized on Affymetrix Human Gene 2.0 ST arrays. Staining and scanning was done according to the Affymetrix expression protocol including minor modifications as suggested in the Encore Biotion protocol. Expression console (v.1.3.0.187, Affymetrix) was used for quality control and to obtain annotated normalized RMA gene-level data (standard settings including median polish and sketch-quantile normalization). Statistical analyses were performed by utilizing the statistical programming environment R (R Development Core Team, 2008) implemented in CARMAweb (Rainer et al., 2006). Genewise testing for differential expression was done employing the (limma) t-test and Benjamini-Hochberg multiple testing correction (FDR <10%). To reduce the background, sets of regulated genes were filtered for average expression >10 in at least one of the three groups. Heatmaps were generated with CARMAweb and GO term and pathway enrichment analyses (p<0.01) were done with GePS (Genomatix). Array data has been submitted to GEO (GSE64248).

(http://www.ncbi.nlm.nih.gov/geo/query/acc.cgi?token=cvwdayyudbydzwj&acc=GSE64248)

Development • Supplementary information

SUPPLEMENTAL REFERENCES

Rainer, J., Sanchez-Cabo, F., Stocker, G., Sturn, A. and Trajanoski, Z. (2006). CARMAweb: comprehensive R- and bioconductor-based web service for microarray data analysis. *Nucleic Acids Res.* **34**, W498–503.

Schmittgen, T. D. and Livak, K. J. (2008). Analyzing real-time PCR data by the comparative C(T) method. *Nat Protoc* **3**, 1101–1108.

Stingl, J., Emerman, J. T. and Eaves, C. J. (2005). Enzymatic dissociation and culture of normal human mammary tissue to detect progenitor activity. *Methods Mol. Biol.* **290**, 249–263.

Wozniak, M. A. and Keely, P. J. (2005). Use of three-dimensional collagen gels to study mechanotransduction in T47D breast epithelial cells. *Biol Proced Online* **7**, 144–161.
